# Orally Administrated *Lactobacillus pentosus* var. *plantarum* C29 Ameliorates Age-Dependent Colitis by Inhibiting the Nuclear Factor-Kappa B Signaling Pathway via the Regulation of Lipopolysaccharide Production by Gut Microbiota

**DOI:** 10.1371/journal.pone.0116533

**Published:** 2015-02-17

**Authors:** Jin-Ju Jeong, Kyung-Ah Kim, Se-Eun Jang, Jae-Yeon Woo, Myung Joo Han, Dong-Hyun Kim

**Affiliations:** 1 Department of Life and Nanopharmaceutical Sciences, Kyung Hee University, 1, Hoegi, Dongdaemun-gu, Seoul, 130–701, Korea; 2 Department of Food and Nutrition, Kyung Hee University, 1, Hoegi, Dongdaemun-gu, Seoul, 130–701, Korea; Sun Yat-sen University Cancer Center, CHINA

## Abstract

To evaluate the anti-inflammaging effect of lactic acid bacteria (LAB) on age-dependent inflammation, we first screened and selected a tumor necrosis factor (TNF)-α and reactive oxygen species (ROS)-inhibitory LAB, *Lactobacillus pentosus* var. *plantarum* C29, among the LABs isolated from fermented vegetables using LPS-stimulated mouse peritoneal macrophages. Oral administration of C29 (2 × 10^9^ CFU/rat) for 8 weeks in aged Fischer 344 rats (age, 16 months) inhibited the expression of the inflammatory markers myeloperoxidase, inducible nitric oxide (NO) synthase, cyclooxygenase-2, pro-inflammatory cytokines tumor necrosis factor (TNF)-α and IL-6 and the activation of nuclear factor kappa-light-chain-enhancer of activated B cells (NF-κB), activator protein 1 (AP1), and mitogen-activated protein kinases (MAPKs). Treatment with C29 induced the expression of tight junction proteins ZO-1, occludin, and claudin-1, and reduced intestinal microbial LPS and plasmatic LPS levels and ROS, as well as the *Firmicutes* to *Bacteroidetes* ratio, which is significantly higher in aged rats than in young rats. C29 treatment also reduced plasmatic reactive oxygen species, malondialdehyde, C-reactive protein, and TNF-α, and suppressed expression of senescence markers p16 and p53 in the colon of the aged rats, but increased SIRT 1 expression. Based on these findings, we concluded that C29 treatment may suppress aging-dependent colitis by inhibiting NF-κB, AP1, and MAPK activation via the inhibition of gut microbiota LPS production and the induction of tight junction protein expression.

## Introduction

Aging is an inevitable consequence of processes characterized by age-dependent physiological and functional decline and is hypothesized to be closely associated with chronic inflammatory states [1,2]. Aging is accelerated by redox imbalances between oxidative stress and anti-oxidative mechanisms, which include oxidative alterations in DNA, protein, and other cellular components. Oxidative stress also continuously activates low-grade inflammatory signaling pathways via the redox-sensitive nuclear factor-kappa B (NF-κB) signaling pathway [1,3], leading to the chronic inflammatory diseases colitis and rheumatoid arthritis, and the age-related degenerative diseases Alzheimer’s disease [4–7] and cardiovascular disease [8]. Aging also increases the expression of the inflammatory markers NF-κB and inducible nitric oxide (NO) synthase (iNOS) and the senescence marker p16 in the colon of mice, as well as the LPS content in the colon fluid [1].

Inflammatory bowel disease (IBD), including ulcerative colitis and Crohn’s disease, is a chronic inflammatory disease that predisposes to colorectal cancer [9–11]. Its pathogenic mechanism is considered to be dysregulation of the intestinal immune response to intestinal environmental antigens, such as gut microbiota [12,13]. Normal gastrointestinal microbiota consists of > 1000 bacterial species and is influenced by endogenous and exogenous factors, such as diet, drugs, stress, and aging [12–14]. There are approximately 10^10^ to 10^12^ microbial cells per gram in the colon of humans and animals. Most of the identified microorganisms belong to the *Bacteroidetes* and *Firmicutes* phyla. *Proteobacteria, Fusobacteria*, and *Actinobacteria* phyla comprise less than 10% of the total community [13,14–16]. Of gut microbiota antigens, LPS significantly stimulates an inflammatory response via the CD14/TLR4/MD2 receptor complex in many cell types, such as monocytes, dendritic cells, macrophages and B cells. LPS stimulation also can induce reactive oxygen species (ROS) production via nicotinamide adenine dinucleotide phosphate oxidase activation in immune cells such as neutrophils. Additionally, aging induces the production of fecal LPS in mice and humans [14,17]. Nevertheless, little is known about the molecular mechanism underlying gut microbiota LPS-stimulated aging.

Lactic acid bacteria (LAB) are gram-positive, acid-tolerant, and generally non-sporulating, bacteria that produce lactic acid. LAB are frequently found in yogurt, cheese, kimchi, and the vagina and gastrointestinal microbiota of humans and animals. Most of the LAB, particularly lactobacilli and bifidobacteria, are reported to be beneficial microorganisms, due to their ubiquitous appearance in food and their contribution to the healthy microbiota. Among the LAB, *Lactobacillus* spp. are a genus of gram-positive facultative anaerobic or aerotolerant bacteria. In the gastrointestinal tract of humans, the *Lactobacillus* spp. exhibits various beneficial effects, except in the mouth where they are associated with tooth decay and cavities [18]. *Lactobacillus* spp. reverse the disruptions in indigenous microbiota [19,20], induce non-specific activation of the host’s immune system in humans and animals, and have anti-diabetic [21] and anti-colitic effects [22,23]. However, the anti-inflammaging effect of LAB has been not studied thoroughly in aged animals.

Therefore, to evaluate the effect of LAB on age-dependent inflammation, we screened and selected the tumor necrosis factor (TNF)-α expression-inhibitory strain C29 from LAB isolated from fermented vegetables and investigated its anti-colitic and anti-aging effects in aged Fischer 344 (F344) rats.

## Materials and Methods

### Materials

A limulus amebocyte lysate (LAL) assay kit was purchased from Cape Cod Inc. (E. Falmouth, MA, U.S.A.). Radioimmuno-precipitation assay (RIPA) buffer, tetramethyl benzidine and RPMI1640 were purchased from Sigma (St Louis, MO, U.S.A.). ELISA kits for TNF-α, IL-1β, IL-6, and IL-10 were purchased from R&D Systems (Minneapolis, MN, U.S.A.). DNA isolation kit was purchased from Qiagen (Hilden, Germany). Antibodies for cyclooxygenase-2 (COX-2), iNOS, Forkhead box O3a (FoxO3a), phosphor-FoxO3a, p16, p65, and β-actin were purchased from Santa Cruz Biotechnology (Santa Cruz, CA, U.S.A.). Antibodies for SIRT 1, phosphor-p65, mammalian target of rapamycin (mTOR), phosohor-mTOR, nuclear factor-kappa B inhibitor, α (IκBα), cAMP response element binding protein (CREB), extracellular signal-regulated kinase (ERK), phosphor-CREB, and phosphor-ERK were purchased from Cell Signaling Technology (Beverly, MA, U.S.A.). Other chemicals used were of the highest grade available.

### Preparation of *Lactobacillus* C29

*Lactobacillus* C29 (KCCM10885, Korea Culture Center of Microorganisms, Seoul, Korea) was cultured according to the previously reported method [24]. Briefly, C29 was grown to an optical density between 3 and 4 at 600 nm in MRS broth (10 L), harvested by centrifugation (10,000 × g for 30 min), and washed with phosphate-buffered saline (PBS). The collected cells (2 × 10^9^ CFU/0.1 mL) were suspended in 50 mM sodium bicarbonate buffer containing 1% glucose and were orally administered to rats.

### Animals

All experiments were performed in accordance with the NIH and Kyung Hee University guidelines for University Laboratory Animals Care and Use and were approved by the Committee for the Care and Use of Laboratory Animals in the College of Pharmacy, Kyung Hee University (Permit Number: KHP-2012-11-02).

Male Fischer 344 rats (18 months-old) obtained from Harlan (Indianapolis, IN) and Male C57BL/6 (18–22 g, 6 weeks) supplied from the Central Animal Breeding Center (Seoul, Korea), were provided with water and food ad libitum, and maintained in a ventilated room at an ambient temperature of 22°C ± 1°C with 50% ± 10% humidity and a 12-h diurnal light cycle (lights on 07:00–19:00) for 1 week before the experiment. All behavioral experiments were performed in a room adjacent to the housing room under the same ambient conditions.

For the study on the anti-colitic effect of C29 in aged Fischer 344 rats, C29 (2x10^9^ CFU/rats) or rapamycin, substance used as reference (1 mg/kg/day), was orally administered once a day for 8 weeks.

### Macroscopic score assessment of colitis and colon tissue preparation

The rats were sacrificed 20 h after the final administration of C29. Macroscopic assessment of the colitis grade was scored according to a previously reported scoring system (0, no ulceration and no inflammation; 1, no ulceration and local hyperemia; 2, ulceration without hyperemia; 3, ulceration and inflammation at one site only; 4, two or more sites of ulceration and inflammation; 5, ulceration extending more than 2 cm) [25]. And then the colon was stored at −80°C for immunoblotting and ELISA. For histologic exams, the colons were fixed in 10%-buffered formalin solution, embedded in paraffin, cut into 7-μm sections, stained with hematoxylin-eosin, and then observed under a light microscope.

### Colon tissue preparation

The colons were excised, perfused with ice-cold perfusion solution (0.15 M KCl, 2 mM EDTA, pH 7.4), like the previously reported [25], homogenized in 50 mM Tris-HCl buffer (pH 7.4), and centrifuged at 10,000 × g at 4°C for 30 min and the supernatants used for myeloperoxidase activity, ELISA and immunoblotting assays.

### Assay of myeloperoxidase activity

An aliquot (50 μl) of the colon homogenate supernatant was added to a reaction mixture of 1.6 mM tetramethyl benzidine and 0.1 mM H_2_O_2_ and incubated at 37°C; the absorbance was obtained at 650 nm over time [25]. Myeloperoxidase activity was defined as the quantity of enzyme degrading 1 μmol/mL of peroxide at 37°C and expressed in unit/mg protein. The protein content was assayed by the method of Bradford [26].

### ELISA and immunoblotting

For the ELISA of TNF-α, IL-1β, IL-4, IL-6, and IL-10, the colons were homogenized in 1 ml of ice-cold RIPA lysis buffer containing 1% protease inhibitor cocktail and 1% phosphatase inhibitor cocktail. The lysate was centrifuged (15,000 × g, 4°C) for 15 min, and the supernatant or the macrophage-cultured supernatant was transferred to 96-well ELISA plates. TNF-α, IL-1β, IL-4, IL-6, and IL-10 concentrations were assayed using commercial ELISA kits (Pierce Biotechnology, Inc., Rockford, IL, USA) [25].

For the immunoblot analyses of iNOS, COX2, p-p65, p-IκB, p-ERK, ERK, p-JNK, JNK, p-p38, p38, p-cJun, cJun, and β-actin, the colon homogenate supernatant or the macrophage homogenate supernatant, which were prepared by the collection, homogenization and centrifugation of the cultured macrophages, were used for the immunobloting. The supernatant were subjected to electrophoresis on 8–10% sodium dodecyl sulfate-polyacrylamide gel and then transferred to nitrocellulose membrane. Immunodetection was carried out using an enhanced chemiluminescence detection kit.

### Isolation and culture of peritoneal macrophages

Male C57BL/6 mice were intraperitoneally injected with 2 ml of 4% thioglycolate solution [25]. Mice were sacrificed 4 days after injection and the peritoneal cavities were flushed with 10 ml of RPMI 1640. The peritoneal lavage fluids were centrifuged at 200 × g for 10 min and the cells were resuspended with RPMI 1640 and plated. After incubation for 1 h at 37°C, the cells were washed three times and nonadherent cells were removed by aspiration. Cells were cultured in 24-well plates (0.5 × 10^6^ cells/well) at 37°C in RPMI 1640 plus 10% FBS. The attached cells were used as peritoneal macrophages.

To examine the anti-inflammatory effect of test agents, peritoneal macrophages were incubated in the absence or presence of test agents with 50 ng/ml LPS.

### Determination of LPS

Fecal and plasma endotoxin contents were determined by Limulus amoebocyte lysate (LAL) assay kit (Associates of Cape Cod Inc., U.S.A.) according to manufacturer’s protocol. Briefly, plasma (5 μl) was diluted 1:10 in pyrogen-free water, inactivated for 10 min at 70°C, and incubated with LAL solution for 30 min at 37°C. Addition of reagents led to formation of a magenta derivative that absorbs light at 545 nm. Feces from the rat cecum (100 mg) were placed in 50 mL of PBS in a pyrogen-free tube and sonicated for 1 h on ice. After centrifugation at 400 × *g* for 10 min, the supernatant was collected, sterilized by filtration through a 0.45 μm filter followed by re-filtration through a 0.22 μm filter, and inactivated at 70°C for 10 min. The filtered sonicate (50 μl) was incubated with LAL solution at 37°C for 30 min. Additional reagents led to formation of a magenta derivative that absorbs at 545 nm

### DNA extraction, pyrosequencing, and data analysis

Genomic DNA was extracted from fresh stools using a commercial DNA isolation kit (QIAamp DNA stool mini kit). Amplification of the genomic DNA was performed using barcoded primers, which targeted the V1 to V3 region of the bacterial 16S rRNA gene. Pyrosequencing was performed using a 454 GS FLX Titanium Sequencing System (Roche, Branford, CT), as previously described [23]. Sequence reads were identified using the EzTaxon-e database (http://eztaxon-e.ezbiocloud.net/) on the basis of 16S rRNA sequence data. The number of sequences analyzed, observed diversity richness (operational taxonomic units, OTUs), estimated OTU richness (ACE and Chao1), and coverage in the present pyrosequencing are indicated in S1 Table. 454 pyrosquencing reads have been deposited in the NCBI’s short read archive under accession number SRP049130.

### Determination of inflammaging markers in plasma

Reduced Glutathione (GSH) concentration and reactive oxygen species (ROS) concentration were measured using the GSH detection assay kit (Abcam Inc., Cambridge, MA, U.S.A.) and the ROS assay kit (Cell Biolabs, Inc., San Diego, CA, U.S.A.), respectively. SOD activity was measured spectrophotometrically with the Dojindo SOD Activity Kit-WST (Dojindo Molecular Technologies, Rockville, MD, U.S.A.). Malondialdehyde (MDA) concentration in plasma was determined in terms of thiobarbituric acid reactive substances (TBARS) formation as described by Ohkawa et al. [27]. Plasmatic C-reactive protein (CRP), TNF-α, IL-1β, IL-6, and IL-10 concentrations were assayed using commercial ELISA kits (R & D systems Inc., Minneapolis, MN, U.S.A.) following manufacturer.

### Statistical analysis

All data are expressed as the mean ± standard deviation (SD), with statistical significance analyzed using one-way ANOVA followed by a Student-Newman-Keuls test.

## Results

### Isolation of TNF-α-inhibitory LAB, *Lactobacillus* C29, in LPS-stimulated peritoneal macrophages

**Fig 1:**
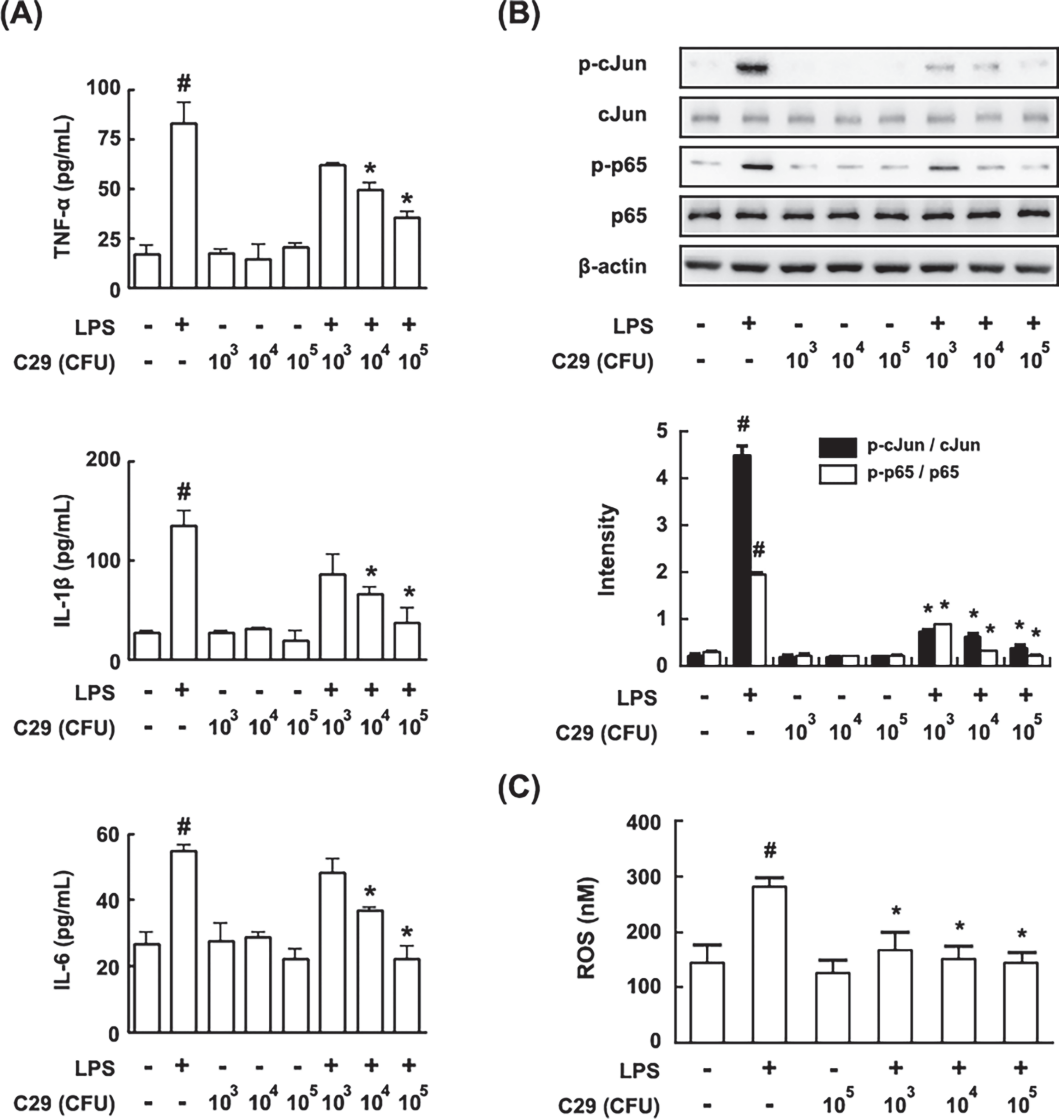
Effect of C29 on the expression of TNF-α, IL-1β, IL-6, and ROS and the activation of NF-κB in peritoneal macrophages stimulated with LPS. The peritoneal macrophages were isolated from mice and incubated with or without LPS (50 ng/mL) in the absence or presence of C29 for 90 min (for activation of c-Jun and NF-κB) or 24 h (expression of TNF-α, IL-1β and IL-6). Protein expression was assayed by ELISA (A) and immunoblotting (B). ROS was assayed by ROS assay kit (C). ^#^, *p* < 0.05 compared with untreated group; *, *p* < 0.05 compared with LPS alone.

To evaluate the effect of anti-inflammatory LAB on age-dependent inflammation, we screened TNF-α expression-inhibitory LAB from collected kimchi LAB strains in LPS-stimulated rat peritoneal macrophages. Of the tested LAB, *Lactobacillus pentosus* var. *plantarum* C29 inhibited LPS-stimulated TNF-α expression most potently (Fig. 1A). Furthermore, C29 inhibited NF-κB and activator protein 1 (AP1) activation, as well as IL-1β and IL-6 expression (Fig. 1A and 1B). Treatment with C29 also decreased the ROS level in LPS-stimulated rat peritoneal macrophages (Fig. 1C).

### Anti-inflammaging effect of C29 in the colon of the aged rats

**Fig 2:**
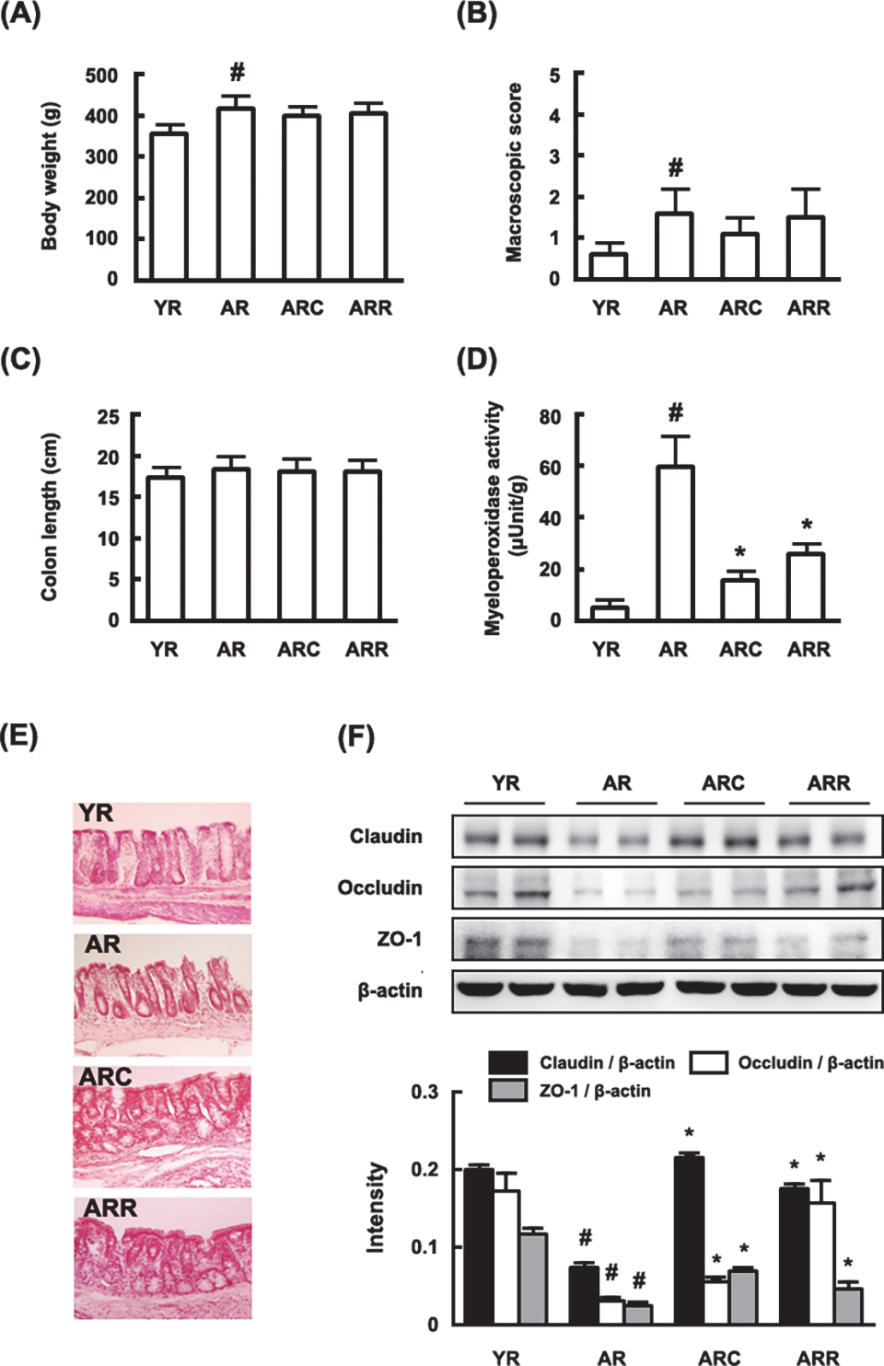
Effect of C29 on body weight (A), macroscopic score (B), colon length (C), intestinal myeloperoxidase activity (D), histology (E), and expression of tight junction proteins (F) in young and aged F344 rats. YR, young rats; AR, aged rats; ARC, aged rats treated with C29, ARC, aged rats treated with rapamycin (n = 10). ^#^, *p* < 0.05 compared with YR; *, *p* < 0.05 compared with AR.

Next, we investigated the anti-inflammaging effect of C29 in the colon of aged F344 rats. In the present study, rapamycin, the inhibitor of mammalian TOR (mTOR) used as reference [28]. Although the colon length of the aged mice was significantly longer than in the young rats, unlike chemical-induced colitis [27], the macroscopic inflammation score of the colon of aged rats was higher than that of the young (Fig. 2). The myeloperoxidase activity of the aged rats were higher than those of the young rats (Fig. 2D). Histologic examination of the colon in the aged rats showed dense infiltration of the superficial layers of the mucosa and epithelial cell disruption by ulcerations (Fig. 2E). Treatment with C29 or rapamycin in the aged rats suppressed edema and epithelial cell disruption, as well as myeloperoxidase activity compared with those of the untreated aged rats. Furthermore, treatment of C29 or rapamycin increased the expression of the colonic tight junction proteins claudin, occludin, and ZO-1, which are typically suppressed in the colon of the aged rats, relative to that in young rats (Fig. 2F).

**Fig 3:**
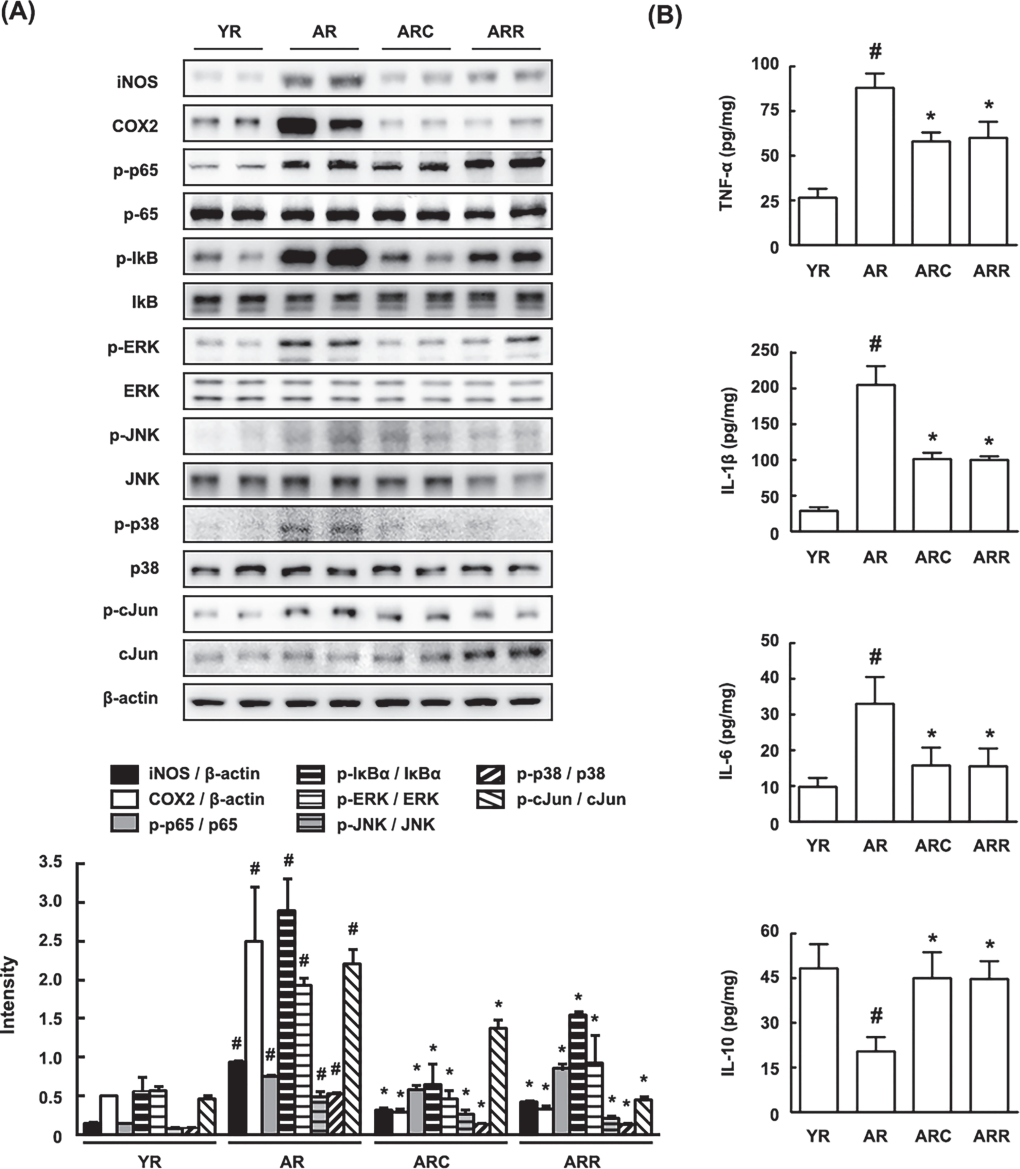
Effect of C29 on the expression of inflammatory markers COX2 and iNOS and proinflammatory cytokines TNF-α, IL-1β and IL-6 and the activation of their transcription factors NF-κB, AP1, MAPKs in the colon of young and aged F344 rats. Protein expression was assayed by immunoblotting (A) and ELISA (B). YR, young rats; AR, aged rats; ARC, aged rats treated with C29, ARR, aged rats treated with rapamycin (n = 10). ^#^, *p* < 0.05 compared with YR; *, *p* < 0.05 compared with AR.

Next, we measured the expression of the inflammatory markers COX2 and iNOS and proinflammatory cytokines TNF-α, IL-1β and IL-6 and the activation of their transcription factors NF-κB, AP1, and MAPKs, which are generally activated by ROS and LPS [31], in the colon of young and aged rats. The expression levels of COX-2, iNOS, TNF-α, IL-1β and IL-6 were higher in aged rats than in young rats (Fig. 3A and B). The activation of NF-κB and the levels of AP1 and MAPKs in the aged rats also increased more potently than those in young rats. Treatment with C29 or rapamycin in the aged rats inhibited the expression of COX2, iNOS, and proinflammatory cytokines TNF-α and IL-1β, as well as the activation of NF-κB, AP1, and MAPKs.

**Fig 4:**
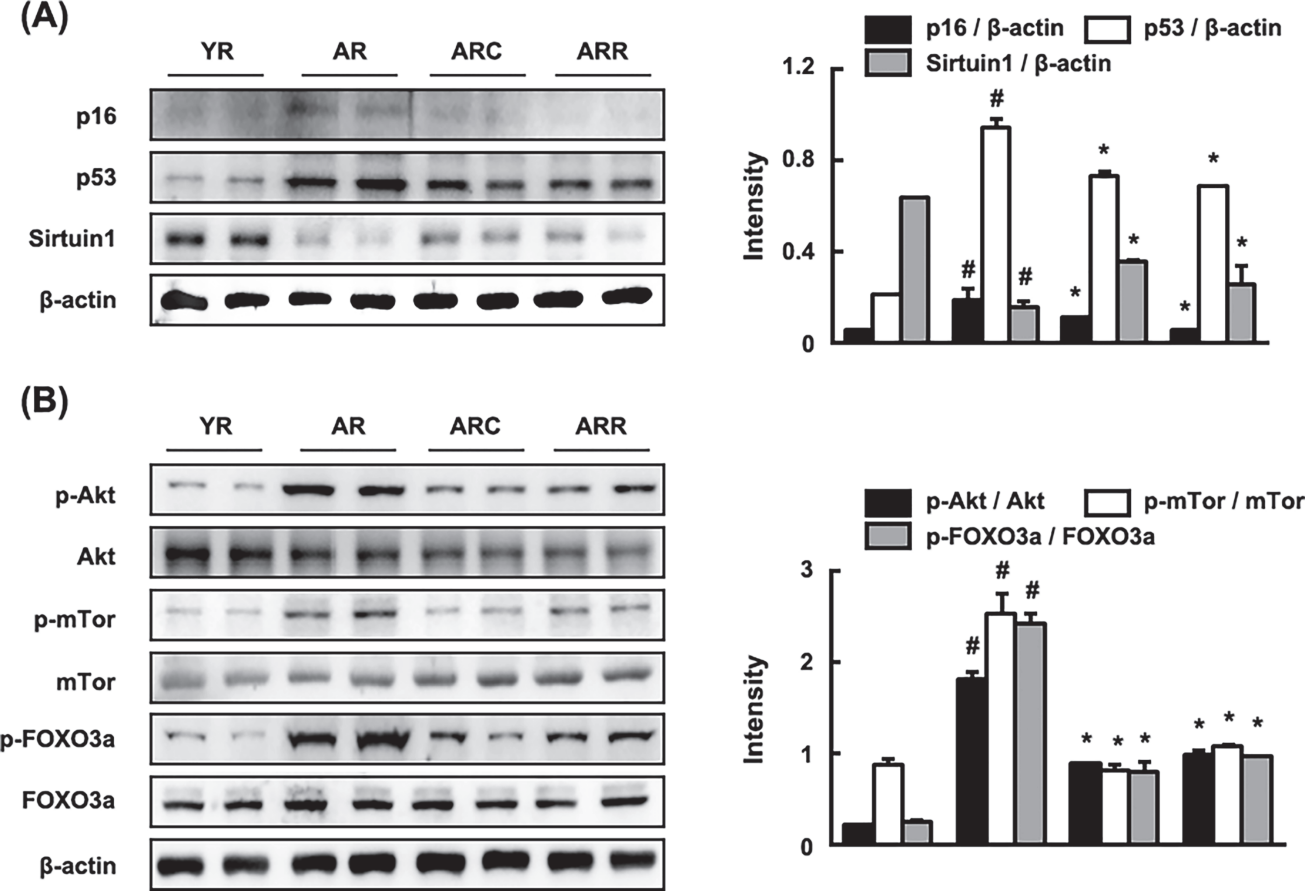
Effect of C29 on the expression levels of senescence markers p16, p53 and SIRT 1 (A) and cell survival signaling molecules Akt, mTOR and FOXO3a (B) in young and aged F344 rats. YR, young rats; AR, aged rats; ARC, aged rats treated with C29, ARR, aged rats treated with rapamycin (n = 10).

Next, we measured the expression levels of senescence markers p16, p53, and SIRT 1 in rats (Fig. 4). The expression of senescence markers p16 and p53 was significantly higher in the colon of 18 months-aged rats than in young rats (4 and 6 months-old rats), but SIRT 1 expression was lower (Fig. 4A). Among cell survival signaling molecules, Akt, mTOR, and FOXO3αwere activated more potently in the colon of 18-month-old rats than in young rats (Fig. 4B). Treatment with C29 or rapamycin suppressed p16 and p53, but increased the expression of SIRT 1. Furthermore, treatment with C29 or rapamycin inhibited the phosphorylation of Akt, mTOR and FOXO3a.

### Effect of C29 on gut microbiota composition in aged rats

Next, we investigated the difference in the composition of gut microbiota between young and aged rats by 16S rRNA pyrosequencing. Bacterial richness and diversity between the various feces samples of rats were not different, as described by the number of sequences analyzed, estimated operational taxonomic unit (OTU) richness, and coverage (S1 Table).

**Fig 5:**
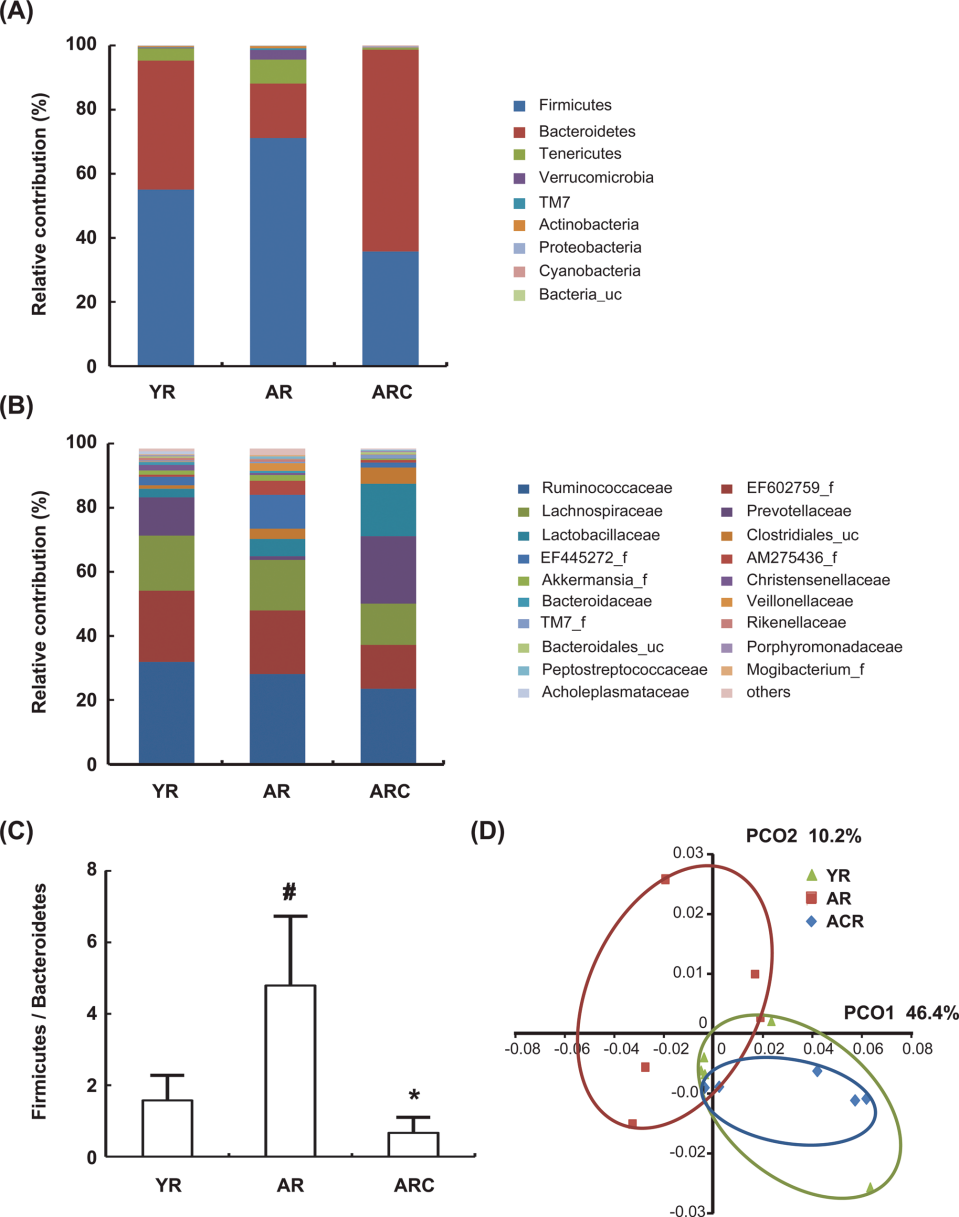
Effect of C29 on the composition of gut microbiota. Taxonomy compositions: (A) phylum and (B) family levels are shown. Genomic DNA was extracted from the fecal samples taken from young, aged or C29-treated aged F344 rats and analyzed for bacterial composition by pyrosequencing of the bacterial 16S rRNA fragments (n = 5). (C) The *Firmicutes* to *Bacteroidetes* ratio (n = 5). (D) Principal coordinate analysis (PCoA) plot. The plot shows the clustering pattern among young, aged, or C29-treated aged F344 rats based on weighted pairwise Fast UniFrac analysis (n = 5). YR, young rats; AR, aged rats; ARC, aged rats treated with C29, ARC; ^#^, *p* < 0.05 compared with YR; *, *p* < 0.05 compared with AR.

By taxonomy-based analysis, aging induced a significant modulation of the populations of the dominant gut microbiota as compared to that of young rats. At the phylum level, aging resulted in a significant increase in *Firmicutes* and *Tenericutes*, as well as a reduction in *Bacteroidetes*, which led to an increase in the *Firmicutes* to *Bacteroidetes* ratio in the gut microbiota (Fig. 5A and C). Treatment with C29 in aged rats significantly decreased the *Firmicutes* to *Bacteroidetes* ratio. At the family level, *EF445272_f* and *AM275436_f* were enriched in the aged rats as compared to the levels in young rats, while *Prevotellaceae* decreased (Fig. 5B). Treatment with C29 increased the number of *Prevotellaceae*, but decreased the numbers of *EF445272_f* and *AM275436_f*. At the genus level, aging resulted in a decrease in *Bacteroides* and *HM123280_g* and an increase in *EU381725_g*, *Clostridiales_uc_g*, and *Eubacterium _g8*. C29 treatment in aged rats increased the numbers of *Bacteroides* and *Prevotella* (S1 and S2 Figs.). In addition, at the species level, aging decreased the numbers of *Bacteroides uniformis*, *EF406830_s*, *EF406459_s*, but increased the numbers of *DQ777952_s*, *4P003470_s*, and *AM275436_f_uc_s*. However, C29 treatment increased the numbers of *Bacteroides*, *Bacteroides acidifaciens*, and *EF406459_s*.

Next, we processed all these sequences to match the length and position of gut microbiota 16S rRNA gene sequences, computed all pair-wise distances among young, aged, and C29-treated aged rats, and performed principal coordinate analysis (PCoA) (Fig. 5D). The gut microbial community of C29-treated aged rats was clustered between young and aged group communities, indicating partial reversal of the aged group community to the young group community. The maximum variations were 42.1% (PCO1) and 12.10% (PCO2). These results suggest that C29 may restore aging-disturbed gut microbiota composition and LPS production to those of the young rats.

### Anti-imflammaging effect of C29 in the plasma of aged F344 rats

Next, we measured markers of inflammation in the blood of the young and aged rats (Table 1). The plasmatic levels of ROS, MDA, CRP and the proinflammatory cytokines TNF-α and IL-6 in the aged rats were significantly higher than those in young rats. However, plasmatic GSH, superoxide dismutase and IL-4 levels in aged rats were lower than those in young rats, as previously reported [1]. Oral administration of C29 or rapamycin increased GSH levels, but reduced ROS, CRP and MDA levels. Furthermore, treatment with C29 or rapamycin reduced plasma levels of TNF-α and IL-6, but did not affect IL-4 level.

**Table 1:** Plasmatic parameters in young, aged, and C29-treated aged F344 rats.

	YR	AR	ARC	ARR
GSH (μM)	6.6±2.4	5.0±0.7	6.2±1.6	6.5±2.2
ROS (μM)	4679.8±111.2	6164.5±2259.1^#^	5471.8±1990.7	5991.4±1517.3
SOD (U/mL)	2.6±0.1	2.3±0.2^#^	2.5±0.5	2.7±0.4
MDA (nmol/mL)	26.6±6.4	58.4±11.2^#^	46.3±17.9	37.5±17.5
CRP (pg/mL)	304.4±171.0	755.5±150.6^#^	352.6±97.4*	446.4±96.6*
TNF-α (pg/mL)	28.6±13.6	100.1±12.7^#^	46.1±30.1*	46.0±34.9#
IL-1β (pg/mL)	12.3±6.1	169.0±61.0^#^	62.3±22.8*	63.6±29.4*
IL-4 (pg/mL)	50.7±12.4	34.4±9.6^#^	35.9±9.2	35.7±6.4
IL-6 (pg/mL)	7.8 ±7.3	21.7±11.6^#^	13.6±10.4	14.4±10.3
IL-10 (pg/mL)	45.5±14.3	28.1±19.9^#^	37.3±16.3*	39.1±15.7

YR, young rats; AR, aged rats; ARC, aged rats treated with C29, ARR, aged rats treated with rapamycin

^#^ *p*< 0.05 compared with YR;

*, *p*< 0.05 compared with AR.

**Fig 6:**
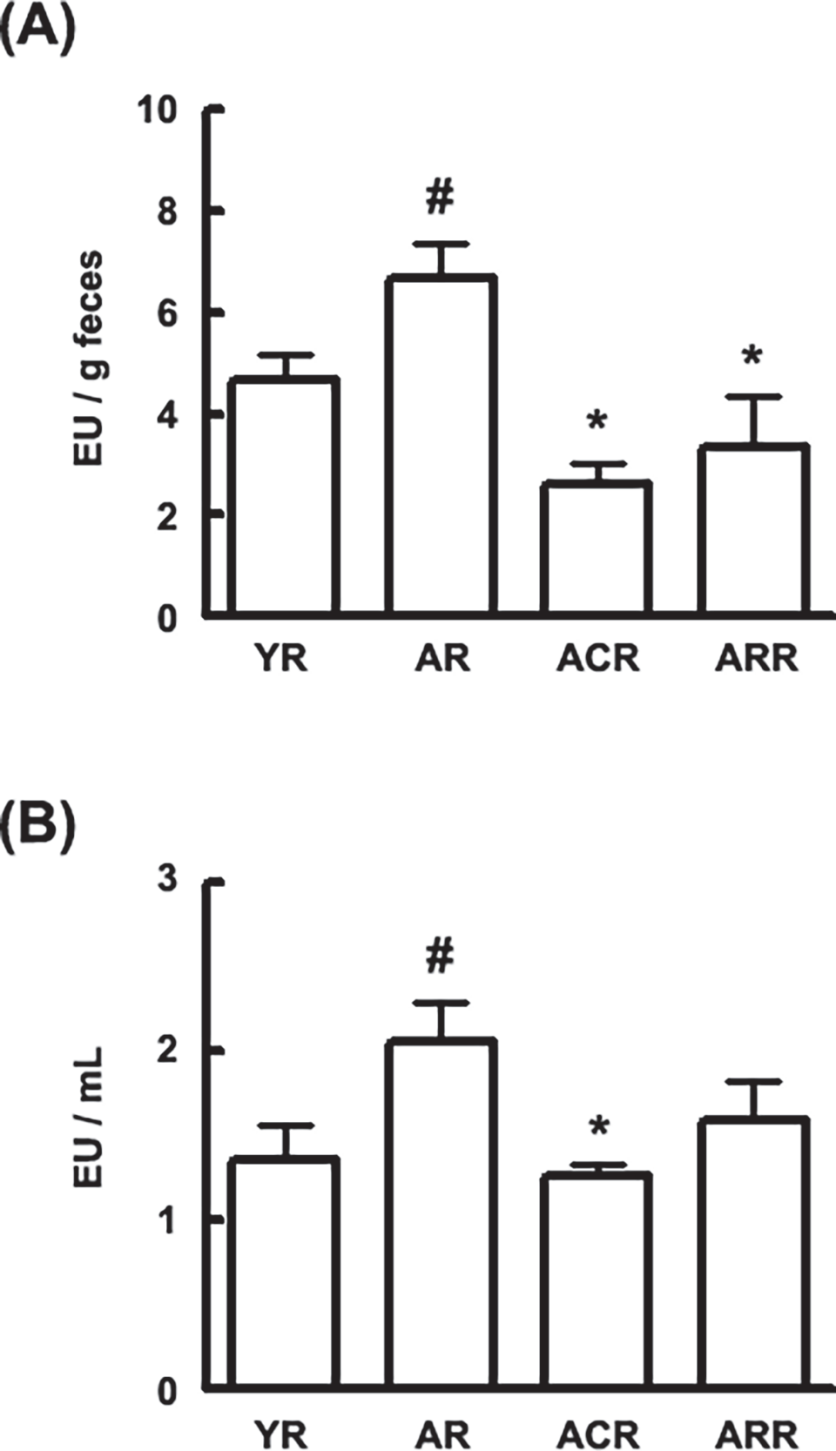
Effect of C29 on the fecal and plasmatic LPS concentrations in young and aged F344 rats. LAL assay was used to measure (A) the fecal and (B) plasmatic endotoxin concentrations. All values are indicated as the mean ± SD (n = 10). YR, young rats; AR, aged rats; ARC, aged rats treated with C29, ARR, aged rats treated with rapamycin (n = 10). ^#^, *p* < 0.05 compared with YR; *, *p* < 0.05 compared with AR.

Additionally, to understand the role of gut microbiota LPS production in inflammaging, we measured fecal and plasmatic LPS concentrations in young and aged rats (Fig. 6). Fecal and plasmatic LPS concentrations were significantly higher in the aged rats than in the young. However, the oral administration of C29 suppressed gut microbiota and plasmatic LPS levels whereas rapamycin treatment decreased plasmatic LPS level in the aged rats.

## Discussion

All surfaces of the human body, including the skin, mucosal surfaces, and the genital and gastrointestinal tracts are occupied by habitat-specific microorganisms. During adult life, the gut microbiota mainly consists of *Bacteroidetes* and *Firmicutes* phyla [14,15]. The community composition of gut microbiota could be influenced by host genetics, health, diet, aging, and probiotics [22,29,30]. Mitsuoka reported that the levels of *Bifidobacterium* were lower in elderly people than in younger adults, whereas the levels of *C*. *perfringens*, lactobacilli, and enterococci increased [31]. Hopkins *et al*. found that *Bifidobacterium* and *Lactobacillus* levels were lower in elderly people than in younger adults, whereas *Bacteroides* and *Eubacterium* levels were the same [32]. Health-promoting bacteria such as bifidobacteria have been thought to decrease with advancing age [33,34]. Nevertheless, levels of facultative anaerobes, including streptococci, staphylococci, enterococci, and enterobacteria, were higher in the elderly individuals than in younger adults. Among them, gram-negative bacteria produce bacterial endotoxins such as LPS, which induces TNF-α and IL-1β [4–6]. Gut microbiota composition is influenced by high-fat diets or aging, which increases LPS-producing bacterial growth, LPS, and ROS production in the intestine [35, 36]. In the present study, C29 potently inhibited the levels of ROS both in LPS-stimulated rat peritoneal macrophages and in blood, as well as gut microbiota LPS production and its absorption into the blood in rats. Furthermore, C29 ameliorated aging-dependent reduction of colonic tight junction protein expression, which regulates the LPS absorption from the gastrointestinal tract into the blood [37]. Therefore, the suppression of tight junction protein expression could accelerate the absorption of LPS from the intestine to the blood [38]. Here, we also found that plasmatic and fecal LPS levels in the aged rats were significantly higher than in the young. These results suggest that the long-term stimulation of these stresses, such as LPS and ROS, activates inflammatory signaling pathways continuously, leading to chronic inflammatory diseases, such as colitis, and consequently intestinal bacterial LPS could be absorbed in the aged rats more easily than in young. Thus, the gastrointestinal bacterial endotoxin LPS is able to increase plasma TNF-α and IL-1β levels by the activation of the low-grade inflammatory signaling pathways, leading to chronic inflammatory diseases such as colitis and rheumatism. Aging therefore attenuates immune responses and disturbs gut microbiota composition, leading to chronic inflammatory diseases, as well as many age-related degenerative diseases. Of these, LPS-stimulated chronic inflammatory diseases may stimulate metabolic diseases such as obesity. To investigate the effect of LAB on inflammation, we screened TNF-α production-inhibitory LAB in LPS-stimulated macrophages. Among the tested LAB, C29 inhibited NF-κB activation most potently. Oral administration of C29 suppressed the expression of colitic markers and proinflammatory cytokines TNF-α, IL-1β, and IL-6 and activity of myeloperoxidase, as well as the activation of NF-κB, IκBα, SIRT 1 and FOXO3α.

The phenotype of tissue macrophages reflects their local metabolic and immune microenvironment [39]. Macrophages are categorized into phenotypic subtypes (M1 and M2) based on the gene expression induced in response to cytokines: classically activated (M1) macrophages and alternatively activated (M2) macrophages characteristically secrete pro-inflammatory and anti-inflammatory cytokines, respectively. In the present study, the mRNA expression levels of M1 macrophage markers such as TNF-α and IL-6 were significantly higher in the colons of aged rats than in those of young rats whereas the mRNA expression level of mannose receptor C1 (Mrc-1), a M2 macrophage marker, was lower in the colons of aged rats than in those of young rats. Interestingly, treatment with C29 decreased the mRNA expression of TNF-α and IL-6 whereas increased those of Mrc-1 in the colon of aged rats (S3 Fig.) suggesting that C29 may restore the disturbed balance between M1 and M2 macrophages induced by aging.

Treatment with C29 also restored the ratio of *Firmicutes* to *Bacteroidetes* in gastrointestinal microbiota of the aged rats, of which *Firmicutes* to *Bacteroidetes* ratio is higher than in the young rats. Treatment with C29 also induced the expression of colonic tight junction proteins in the aged rats. Furthermore, treatment with C29 inhibited plasmatic and fecal LPS production, as well as iNOS and COX-2 expression. These results suggest that treatment with C29 may ameliorate colitis in the aged rats by inhibiting the activation of NF-κB, AP1, and MAPKs.

Based on these findings, we conclude that the inhibition of gut microbiota LPS production and the induction of tight junction proteins by C29 might suppress aging-dependent inflammatory diseases such as colitis.

### RNA extraction and real-time PCR

Total RNA was extracted from colon with the RNeasy Mini kit (Qiagen, Valencia, CA) and reverse transcription was performed with 2 μg of total RNA. Real-time polymerase chain reaction (RT-PCR) was performed using the Takara Thermal Cycler Dice (Takara, Japan).

## Supporting Information

S1 Fig Difference in the composition of fecal bacterial genera isolated from young, aged or C29-treated aged F344 rats. (TIF)

S2 Fig Difference in the composition of fecal bacterial species isolated from young, aged or C29-treated aged F344 rats. (TIF)

S3 Fig Differential mRNA expression of genes encoding M1 and M2 macrophage markers in the colon. All values are indicated as the mean ± SD (n = 10). YR, young rats; AR, aged rats; ARC, aged rats treated with C29, ARR, aged rats treated with rapamycin (n = 10). Mrc-1, mannose receptor C1; Arg-1, arginase-1, ^#^, *p* < 0.05 compared with YR; *, *p* < 0.05 compared with AR.
(TIF)

S1 Table Number of sequences analyzed, operational taxonomic units (OTUs), estimated OTU richness (abundance-based coverage estimator (ACE) and Chao1), and coverage. (DOCX)
