# Correction: Orally Administrated *Lactobacillus pentosus* var. *plantarum* C29 Ameliorates Age-Dependent Colitis by Inhibiting the Nuclear Factor-Kappa B Signaling Pathway via the Regulation of Lipopolysaccharide Production by Gut Microbiota

**DOI:** 10.1371/journal.pone.0142521

**Published:** 2015-11-12

**Authors:** Jin-Ju Jeong, Kyung-Ah Kim, Se-Eun Jang, Jae-Yeon Woo, Myung Joo Han, Dong-Hyun Kim

There is an error in the units for fecal LPS shown in Fig 6A. The correct units are EU per microgram (EU/ μg) feces. Here we provide a corrected Fig 6.

**Fig 6:**
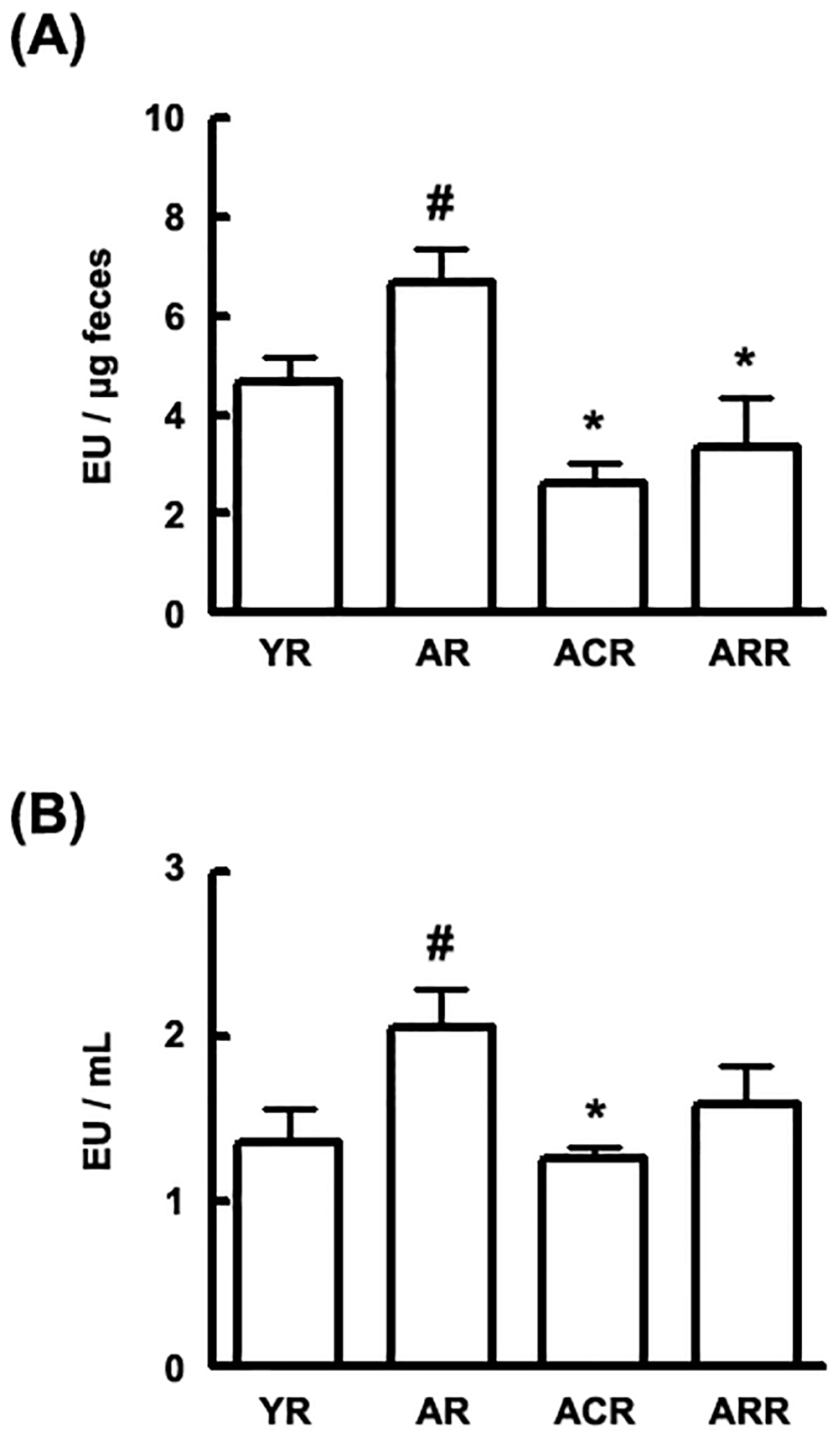
Effect of C29 on the fecal and plasmatic LPS concentrations in young and aged F344 rats. LAL assay was used to measure (A) the fecal and (B) plasmatic endotoxin concentrations. All values are indicated as the mean ± SD (n = 10). YR, young rats; AR, aged rats; ARC, aged rats treated with C29, ARR, aged rats treated with rapamycin (n = 10). ^#^, *p* < 0.05 compared with YR; *, *p* < 0.05 compared with AR.

Additionally, the text in the Results section accompanying Fig 6 incorrectly states that rapamycin treatment decreased plasmatic LPS level in aged rats. This text should be replaced with the following corrected text: "Fecal and plasmatic LPS concentrations were significantly higher in the aged rats than in the young. However, the oral administration of C29 suppressed gut microbiota and plasmatic LPS levels whereas rapamycin treatment decreased fecal LPS level in the aged rats."

The authors apologize for any inconvenience these errors may have caused.
