# Supplementary figures and images for: Orally Administrated Lactobacillus pentosus var. plantarum C29 Ameliorates Age-Dependent Colitis by Inhibiting the Nuclear Factor-Kappa B Signaling Pathway via the Regulation of Lipopolysaccharide Production by Gut Microbiota
Source: PLoS One. 2015 Feb 17;10(2):e0116533. doi: 10.1371/journal.pone.0116533 (PMC4331539; doi:10.1371/journal.pone.0116533)

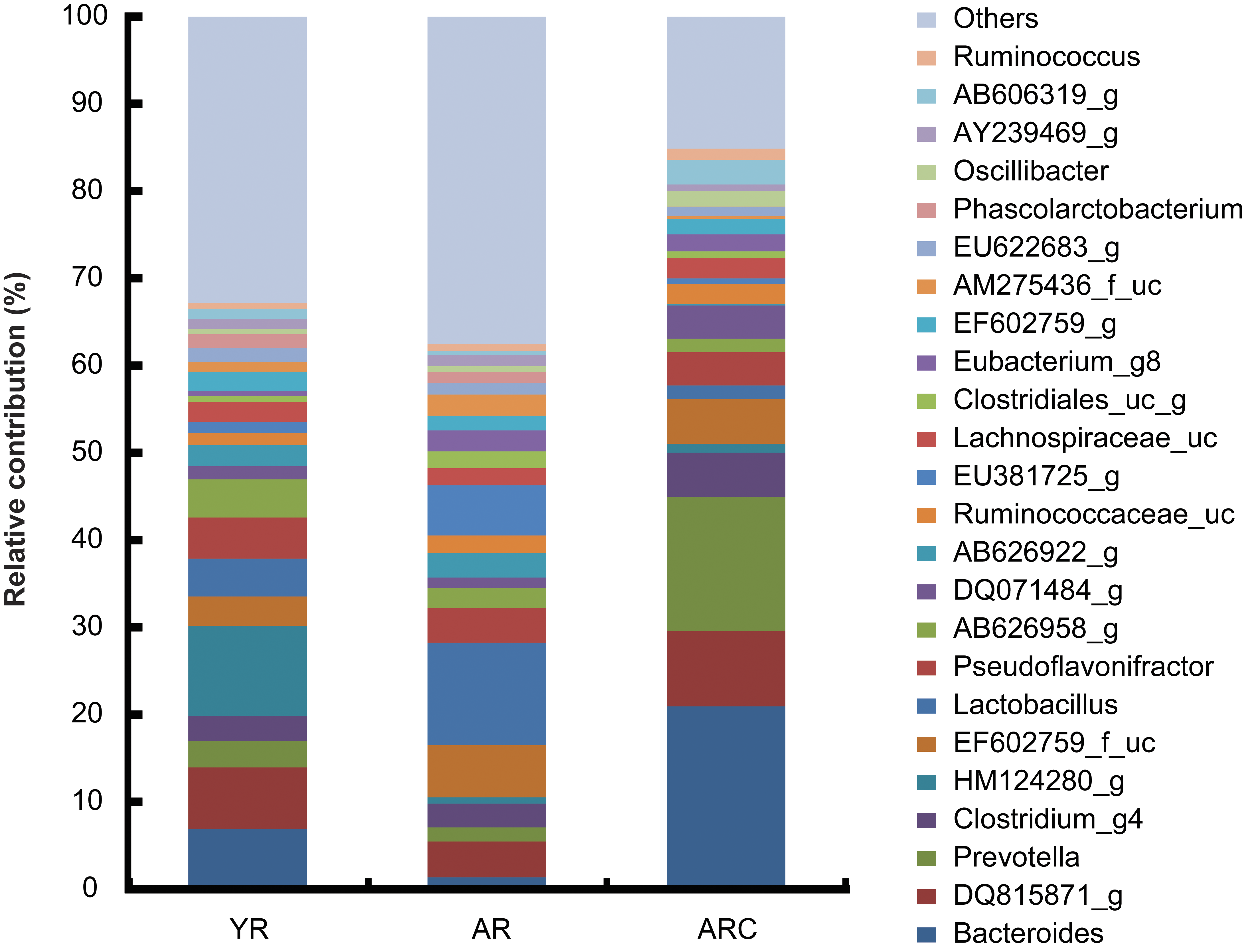

Supplement: S1 Fig — (TIF) [file pone.0116533.s001.tif]

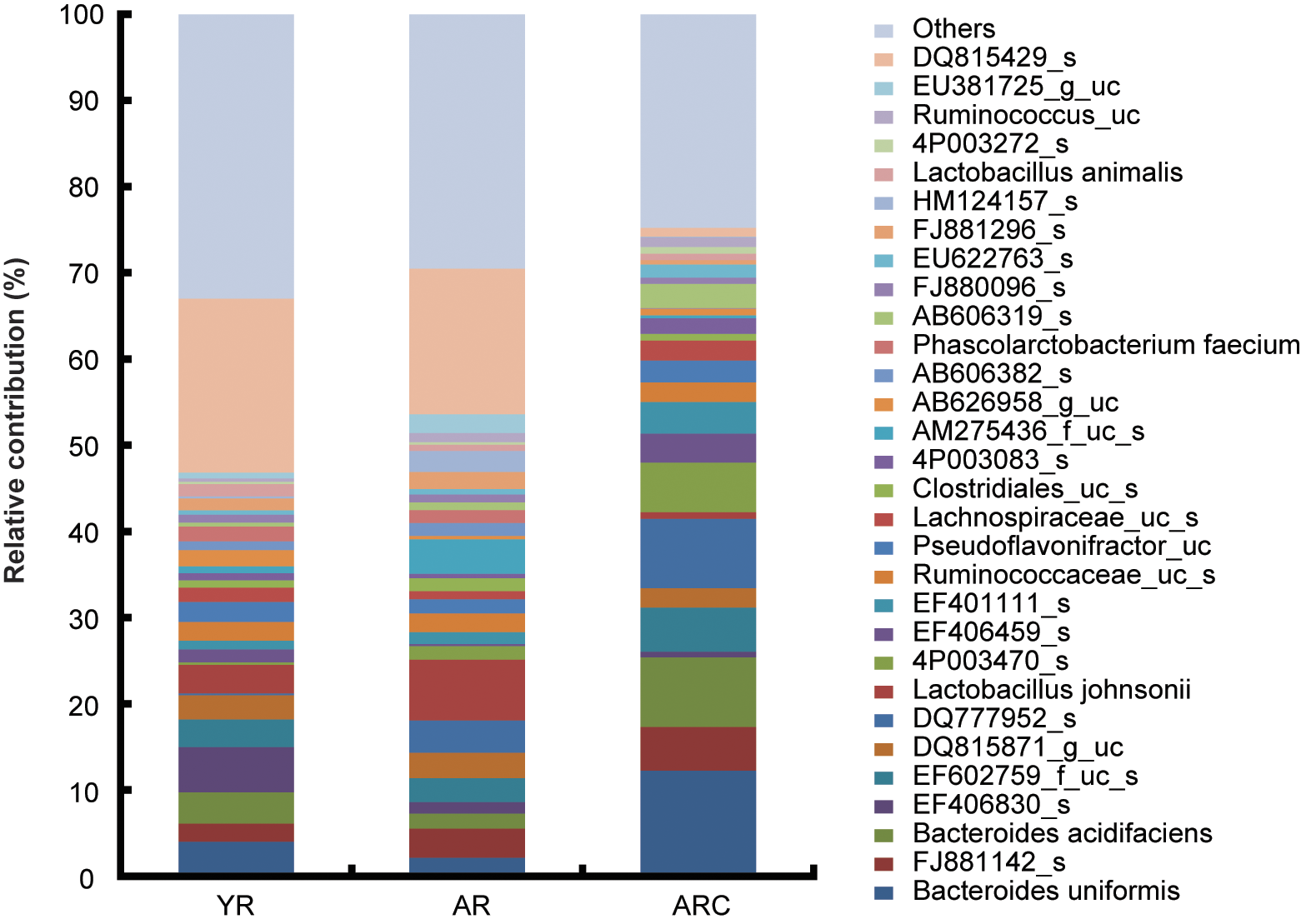

Supplement: S2 Fig — (TIF) [file pone.0116533.s002.tif]

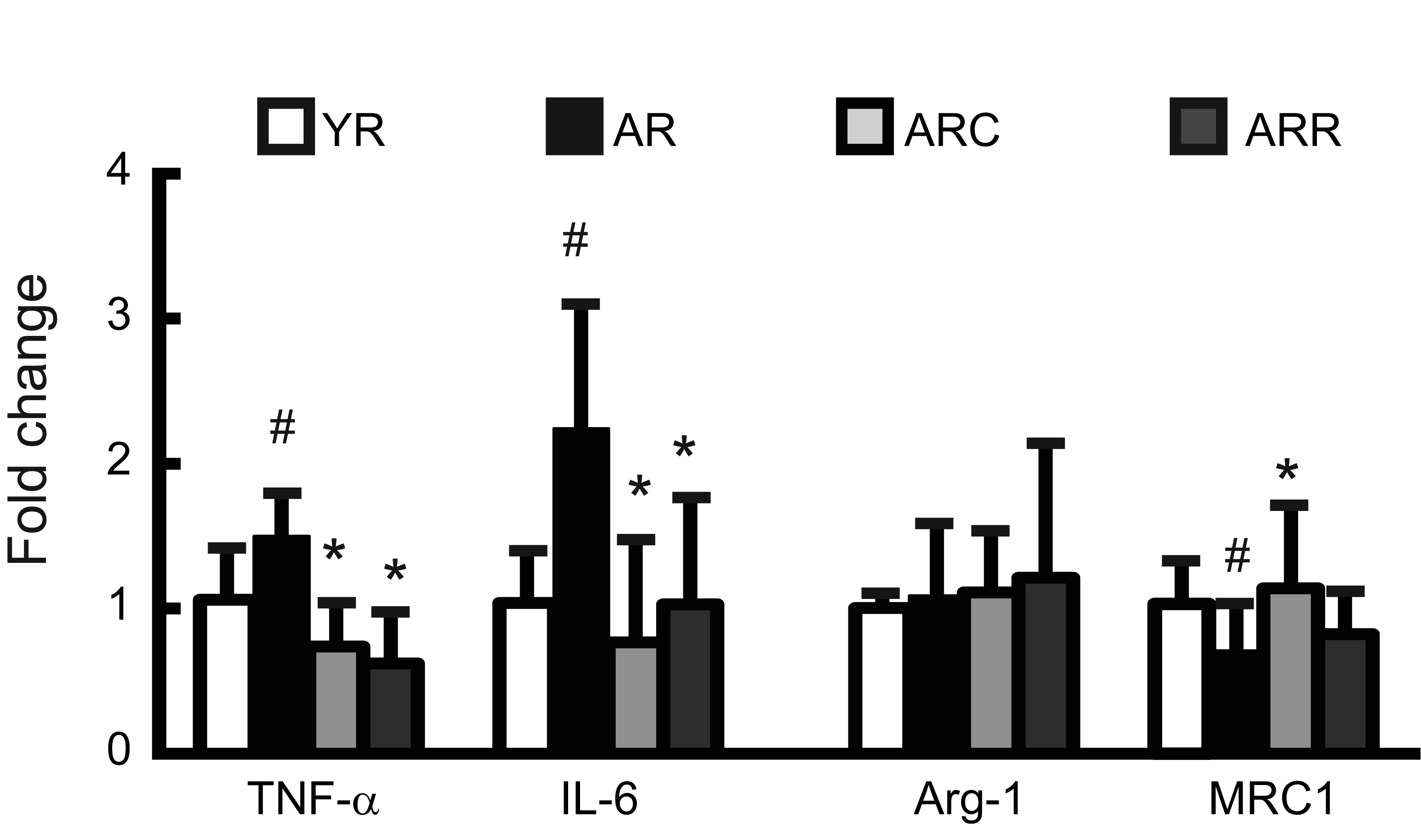

Supplement: S3 Fig — All values are indicated as the mean ± SD (n = 10). YR, young rats; AR, aged rats; ARC, aged rats treated with C29, ARR, aged rats treated with rapamycin (n = 10). Mrc-1, mannose receptor C1; Arg-1, arginase-1, #, p < 0.05 compared with YR; *, p < 0.05 compared with AR. (TIF) [file pone.0116533.s003.tif]
