# Supplementary material for: Orally Administrated Lactobacillus pentosus var. plantarum C29 Ameliorates Age-Dependent Colitis by Inhibiting the Nuclear Factor-Kappa B Signaling Pathway via the Regulation of Lipopolysaccharide Production by Gut Microbiota
Source: PLoS One. 2015 Feb 17;10(2):e0116533. doi: 10.1371/journal.pone.0116533 (PMC4331539; doi:10.1371/journal.pone.0116533)
Supplement: S1 Table — (DOCX) [file pone.0116533.s004.docx]

Table S1. Number of sequence analyzed, observed diversity richness (OTUs), estimated OTU richness (ACE and Chao1), and coverage

|  | Total reads | OTUs | Ace | Chao1 | Goods Coverage |
| --- | --- | --- | --- | --- | --- |
| YR1 | 4774 | 951 | 2277.37 | 1712.36 | 0.90 |
| YR2 | 4488 | 663 | 1429.81 | 1086.16 | 0.93 |
| YR3 | 5960 | 716 | 1531.25 | 1147.46 | 0.94 |
| YR4 | 4866 | 701 | 1591.05 | 1314.09 | 0.93 |
| YR5 | 4764 | 372 | 804.32 | 651.49 | 0.97 |
| Mean±SD | 4970.4±571.0 | 680.6±206.4 | 1526.8±524.1 | 1182.3±384.2 | 0.93±0.02 |
| AR1 | 4817 | 816 | 1990.60 | 1410.95 | 0.91 |
| AR2 | 5266 | 850 | 1983.93 | 1472.54 | 0.92 |
| AR3 | 5222 | 611 | 1179.67 | 951.74 | 0.95 |
| AR4 | 4874 | 523 | 1187.32 | 895.04 | 0.95 |
| AR5 | 5139 | 664 | 1546.30 | 1144.06 | 0.94 |
| Mean±SD | 5063.6±205.2 | 692.8±138.1 | 1577.6±402.3 | 1174.9±261.4 | 0.93±0.02 |
| ARC1 | 5633 | 201 | 354.90 | 329.38 | 0.99 |
| ARC2 | 7061 | 202 | 280.01 | 269.03 | 0.99 |
| ARC3 | 3963 | 880 | 2591.03 | 1843.46 | 0.87 |
| ARC4 | 5050 | 776 | 2150.04 | 1383.57 | 0.92 |
| ARC5 | 6660 | 274 | 407.17 | 395.33 | 0.98 |
| Mean±SD | 5673.4±1246.4 | 466.6±333.3 | 1156.6±1120.0 | 844.2±722.3 | 0.95±0.05 |

The cutoff value of phylotype is equal to or greater than 97% similarity. All values are indicated as the mean±SD. YR, young rats; AR, aged rats; ARC, aged rats treated with C29
